# Increased Risk of Serious Non-AIDS-Related Events in HIV-Infected Subjects on Antiretroviral Therapy Associated with a Low CD4/CD8 Ratio

**DOI:** 10.1371/journal.pone.0085798

**Published:** 2014-01-30

**Authors:** Sergio Serrano-Villar, María Jesús Pérez-Elías, Fernando Dronda, José Luis Casado, Ana Moreno, Ana Royuela, José Antonio Pérez-Molina, Talia Sainz, Enrique Navas, José Manuel Hermida, Carmen Quereda, Santiago Moreno

**Affiliations:** 1 Department of Infectious Diseases, Institute for Health Research (IRICYS), University Hospital Ramón y Cajal, Madrid, Spain; 2 CIBER, Epidemiología y Salud Pública, Clinical Biostatistics Unit, University Hospital Ramón y Cajal, IRYCIS, Madrid, Spain; 3 Laboratory of Molecular Immune Biology, University Hospital Gregorio Marañón, Madrid, Spain; University of California, Davis, United States of America

## Abstract

**Background:**

A low CD4/CD8 ratio has been identified in the general population as a hallmark of inmmunosenescence and a surrogate of all-cause mortality. We aimed to investigate in treated HIV-infected individuals the relationship between the CD4/CD8 ratio and serious non-AIDS events.

**Methods:**

Case-control study within a prospective hospital-based cohort of HIV-infected subjects during at least one year of ART-mediated viral suppression. Cases were patients with serious non-AIDS events (non-AIDS malignancies, cardiovascular disease, and end-stage kidney disease), and controls individuals who did not developed non-AIDS events during follow-up. Data were analyzed using ROC analysis and multivariate logistic regression. Conditional logistic regression was performed in 200 cases/controls matched by age, sex, nadir CD4 and proximal CD4 counts.

**Results:**

We analyzed 407 subjects (109 cases, 298 controls). The CD4/CD8 ratio was lower in cases (0.44 vs. 0.70, *P*<0.0001), with higher discriminatory ability for the detection of non-AIDS events than the CD4 count, CD8 count and nadir CD4. Multivariate analyses (adjusted for age, sex, nadir CD4, proximal CD4 count, year of ART initiation and ART duration) confirmed the independent association of a low CD4/CD8 ratio with the risk of non-AIDS morbidity (per CD4/CD8 ratio quartile decrease, OR, 2.9; 95% CI, 1.3–6.2) and non-AIDS mortality (OR, 2.8; 95% CI, 1.5–5.3).

**Conclusions:**

The CD4/CD8 ratio provides additional information to the CD4 counts and nadir CD4 in treated HIV-infected individuals, since it is independently associated with the risk of non-AIDS-related morbidity and mortality. This association is robust and maintained within different subgroups of patients.

## Introduction

Modern antiretroviral therapy (ART) regimens are among the greatest successes of modern medicine and have shifted the prognosis of HIV-infected patients from years to decades. This remarkable improvement in long-term life-expectancy has brought to the scenario a raising concern about the so-called non-AIDS defining illnesses, a group of conditions generally associated with aging, including cardiovascular disease, renal disease, liver disease, neurocognitive disorders, and non-AIDS malignancies [1]–[6]. Overall, these conditions increase morbidity and mortality despite effective ART [7], [8]. The changing clinical picture of HIV infection has led to a shift in management of HIV infection, to the extent that the usefulness of CD4+ T-cell counts in patients who have reached full HIV RNA suppression has come to debate [9].

Persistent immune activation during treated HIV infection is widely accepted as a driver of non-AIDS-associated diseases [10], [11]. Different conditions are thought to fuel immune activation during treated HIV-infection, including increased bacterial translocation due to a chronically injured mucosa-associated lymphoid tissue (MALT), asymptomatic replication of coinfecting pathogens, especially cytomegalovirus (CMV) and residual viral replication in HIV reservoirs [12]–[16]. Remarkably, HIV-infected subjects display different changes in the adaptive immune system that are shared by the elderly, namely “immunosenescence”, a global term used to describe the observed age-associated decline in immune competence [6], and immune activation is widely accepted as the major driving factor of immunosenescence that ultimately yield to disease progression and adverse outcomes [13], including age-associated disease.

Outside HIV infection, a low CD4/CD8 ratio is considered a surrogate marker of immunosenescence and is an independent predictor of all-cause mortality [17]–[20]. Interestingly, a low CD4/CD8 ratio is practically a universal finding in ART naïve patients and remains low in a substantial proportion of patients who present adequate CD4+ T-cell count recovery. Nevertheless, the clinical significance of a failure to normalize the CD4/CD8 ratio under suppressive ART remains obscure. We have previously described in long-term virally suppressed HIV infected subjects a negative correlation between the CD4/CD8 ratio and immune activation and inmmunosenescence [21], [22], and we also observed that is independently associated with surrogate markers of age-associated disease [23]. Hence, we hypothesized that the CD4/CD8 ratio in ART treated patients can provide additional information to the CD4+ T-cell counts and nadir CD4 and be independently associated to non-AIDS associated morbidity and mortality.

## Methods

### Study Design, Participants, Setting and Eligibility

**Figure 1:**
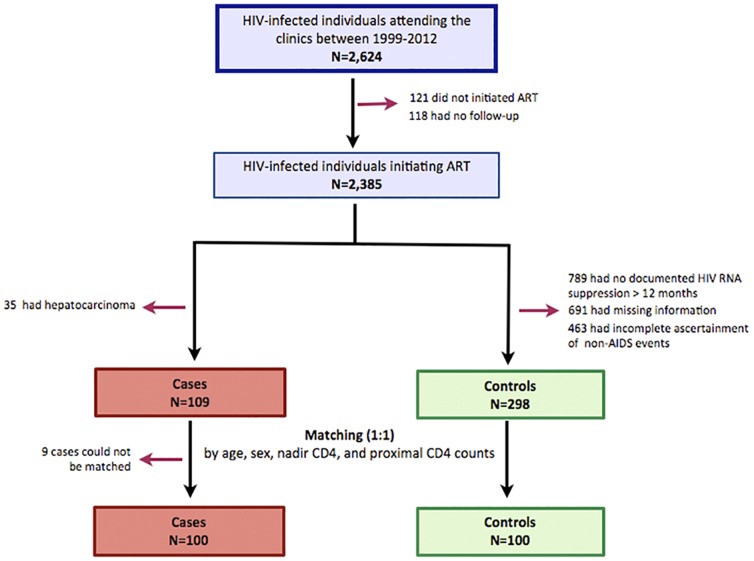
Selection of study participants.

We conducted a case-control study within a clinic-based cohort formed in 1999 of over 2,600 HIV-infected patients receiving care in University Hospital Ramón y Cajal, Madrid. We included HIV-infected adults in this cohort during at least one year of ART-mediated viral suppression. Cases were subjects who developed non-AIDS malignancies, cardiovascular disease, and end-stage chronic kidney disease with available CD4+ and CD8+ T-cell counts measured during routine care and no more than 6 months before the event. For each case, we aimed to identify two ART-suppressed unmatched controls at least one year under viral suppression, in whom the absence of serious non-AIDS events could be ascertained. For the analysis, we used for the last available CD4/CD8 ratio at censoring, which occurred at the time of the event in cases and at the last clinic visit in controls until September 2012. The overall selection of study participants in our study population is described in **Figure 1**. Since 2004, patients who were naïve and started ART at our clinics are enrolled in a nationwide, ongoing prospective multi-center cohort (CoRIS) of HIV-positive subjects. CoRIS is a joint activity of the Research Network of Excellence (AIDS research network, RIS), which incorporates basic scientists, virologists, immunologists, clinicians, epidemiologists and statisticians. Internal quality controls are performed annually and 10% of data are externally audited every year. This database collects demographic and clinical data, risk behaviors, ART history, prior opportunistic diseases, comorbidities, serologic and immunovirological data and specific data on non-AIDS diseases. All subjects from our center in CoRIS database with available CD4/CD8 ratio were included in the study, identifying 348 subjects fulfilling the inclusion criteria. Since we aimed to analyze at least 100 cases, we also searched cases in our general prospective database, in which patients are incorporated since 1999 and therefore, many of them are not enrolled in CoRIS. We carefully reviewed the medical records to ascertain the presence or absence of non-AIDS events. The final study sample included 407 subjects (109 cases and 298 controls).

### Ethics Statement

This study was conformed to the principles or the Declaration of Helsinki and the Good Clinical Practice Guidelines and was approved by our Independent Ethics Committee (Hospital Ramón y Cajal, 28034 Madrid, Spain). All study subjects gave their written informed consent to be included in the database and to analyze the data for investigational purposes.

### Definition on non-AIDS Events

Non-AIDS events involve a broad spectrum of disease, and several definitions to approach the problem in the setting of HIV infection have been used, from subclinical disease such as increased carotid intima-media thickness or osteoporosis to end-stage organ diseases. Since two previous studies in HIV-infected patients specifically described that the CD4/CD8 ratio was associated to cardiovascular disease [24] and Hodgkin lymphoma [25], we used as outcome variable cardiovascular events and non-AIDS defining neoplasias. Data on Hodgkin‘s lymphoma were analyzed separately as Hodgkin‘s lymphoma is not generally considered an age-associated disease [26]. Since chronic kidney disease is usually considered a chronic heart disease risk equivalent [27], we also included subjects with end-stage kidney disease in the definition, according to the stages defined by the Kidney Disease Quality Outcome Initiative [28]. To avoid the potential confounding effect of alcohol abuse and hepatitis C virus (HCV), we excluded patients with end-stage liver disease and hepatocarcinoma (N = 35). Hence, cases were HIV-infected ART-suppressed patients who developed non-AIDS defining malignancies, excluding hepatocarcinoma and non-melanomatous skin cancers, ischemic heart disease (myocardial infarction, percutaneous coronary angioplasty, coronary artery bypass surgery), stroke (ischemic or hemorrhagic), or end-stage kidney disease or. We defined non-AIDS related mortality when the death was caused by any of these conditions.

### Statistical Analysis

Means for variables with a normal distribution were compared using the *t* test and with the Mann-Whitney’s *U* test when distribution of data departed from normality. We compared the CD4/CD8 ratio in cases and controls, and the Spearman correlation coefficient was used to analyse the correlation between the CD4/CD8 ratio and continuous variables. The performance of the CD4/CD8 ratio to discriminate cases and controls was analyzed using the Receiver Operating Characteristic (ROC) curve and calculating the area under curve (AUC). The optimal cutoff was determined by maximizing the sum of sensitivity and specificity. We compared the AUC of the CD4/CD8 ratio with that of the CD4+ T-cell count, CD8+ T-cell count and nadir CD4 using the equality test of ROC areas.

Given that cases and controls showed significant differences in nadir CD4, proximal CD4+ T-cell count and cumulative ART exposure, we attempted to address the potential problem of confounding on the association between non-AIDS events, as dependent variable, and the CD4/CD8 ratio by multivariate logistic regression analysis. Nadir CD4 and cumulative ART exposure were incorporated in the models since they have been previously associated with non-AIDS events [29], [30] as well as with surrogate markers of age-associated disease [31], [32]. Given that the span of entrance in the cohort included a long period, and that individuals starting ART more recently might have received more effective ART, the year of entrance in the cohort was also considered. CD4+ T-cell counts could not be introduced in the models because of collinearity with the CD4/CD8 ratio. Therefore, the model included age, sex, cumulative ART exposure, nadir CD4, year of entrance in the cohort, CD4/CD8 ratio (categorized per quartiles and by the cutoff of 0.4 identified in the ROC analysis) and a potential interaction between the CD4/CD8 ratio and cumulative ART exposure, that was not significant, and was removed from the final model. We specifically analyzed the association between the CD4/CD8 ratio and non-AIDS-associated mortality following the same strategy. To evaluate whether the CD4/CD8 ratio may maintain the association with non-AIDS events in subjects with low nadir CD4, we re-applied the same logistic regression model in the subgroup of patients with low CD4 nadir (<200 cells/µL). Similarly, we explored whether the CD4/CD8 ratio may remain associated with non-AIDS events in subjects with CD4 recovery by reapplying the model to the subgroup of individuals with CD4 count >350 cells/µL.

In order to evaluate whether the CD4/CD8 ratio might provide additional information to the CD4+ T-cell count and the nadir CD4 as a predictor of non-AIDS events, and since these two variables were imbalanced between cases and controls, we performed a nested case-control analysis. We used the Mahapick procedure, a multivariate matching procedure based on a Mahalanobis scoring algorithm, to match cases and controls simultaneously based on age, sex, nadir CD4, and proximal CD4+ T-cell counts, allowing the matching if only one control had complete data. We obtained 200 matched cases and controls in whom we assessed the CD4/CD8 ratio as a predictor of non-AIDS events using conditional logistic regression and adjusting by date of ART initiation and cumulative ART exposure. The null hypothesis was rejected by a type I error <0.05. Statistical analyses were performed using Stata v. 12.0 (StataCorp LP College Station, Texas, USA).

## Results

### General Characteristics of the Study Population and between-groups Comparisons

We analyzed 407 subjects (109 cases and 298 controls), 74% of Caucasian ethnicity and 37% reporting previous injection drug use. Mean age was 43 years (IQR 37–48), median CD4 nadir 194 cells/µL (IQR 74–293), median CD4+ T-cell count 504 cells/µL (IQR 341–661), median CD4/CD8 ratio 0.62 (IQR 0.42–0.88) and mean cumulative ART exposure 4 years (IQR 2–7).

The general characteristics of the study population are summarized in **Table 1**. In the univariate comparisons between cases and controls, no statistically significant differences in sex distribution, geographic origin or risk factors for HIV acquisition were observed. However, patients who developed non-AIDS events were significantly older compared to controls (46 years vs. 41, P<0.0001) and showed significantly longer cumulative ART exposure (9.7 years vs. 3.9, P<0.0001), lower CD4 nadir (124 cells/µL vs. 219, P<0.0001) and lower CD4+ T-cell count (371 cells/µL vs. 536, P = 0.005). These differences disappeared in the subgroup matched by age, gender, nadir CD4 and CD4+ T-cell count, with the exception of cumulative ART exposure, being still longer among cases.

**Table 1:** General characteristics of the study population and in the nested case/control study.

	Study sample			Matched Study		
	N = 407			N = 200		
	Cases	Controls	P value	Cases	Controls	P value
	N = 109	N = 298		N = 100	N = 100	
**Sex, %**			0.468			1.0
Male	77.9	81.1		81.0	81.0	
Female	22.1	18.8		19.0	19.0	
**Age** *	46 (43–49)	41 (34–47)	<0.0001	46 (42–50)	46 (42–50)	1.0
**Geographic Origin, %**			0.301			0.154
Western Europe	70.9	90.5		80.0	93.0	
South America	20.3	–		15.0	–	
Africa	5.8	9.5		3.0	7.0	
Other	3.1	–		2.0	–	
**Risk category, %**			0.098			0.104
Previous IDU	48.4	32.9		47.6	29.2	
Heterosexual	23.8	19.9		28.6	19.8	
Homosexual	21.3	40.8		14.3	42.7	
Other/Unknown	6.5	6.6		9.5	8.3	
**CDC HIV Classification, %**			0.008			0.242
A	27.5	61.2		33.6	42.3	
B	37.2	18.8		31.1	27.4	
C	33.3	19.9		36.3	30.3	
**Cumulative ART exposure (years)** *	9.7 (5.5–13.6)	3.9 (2.3–5.4)	<0.0001	9.8 (5.4–13.5)	4.3 (2.4–6.2)	<0.0001
**CD4 nadir (cell/µL)** *	124 (33–221)	219 (107–303)	<0.0001	121 (40–231)	120 (46–233)	0.9105
**CD4+ count (cell/µL)** *	371 (225–586)	536 (407–678)	<0.0001	404 (260–588)	415 (173–589)	0.689
**CD8+ count (cell/µL)** *	880 (582–1185)	791 (588–1053)	0.007	925 (636–1245)	715 (501–978)	0.002
**CD4/CD8 ratio** *	0.44 (0.30–0.68)	0.70 (0.48–0.94)	<0.0001	0.46 (0.29–0.66)	0.62 (0.40–0.90)	0.001

* Expressed as median (P25–P74).

**Figure 2:**
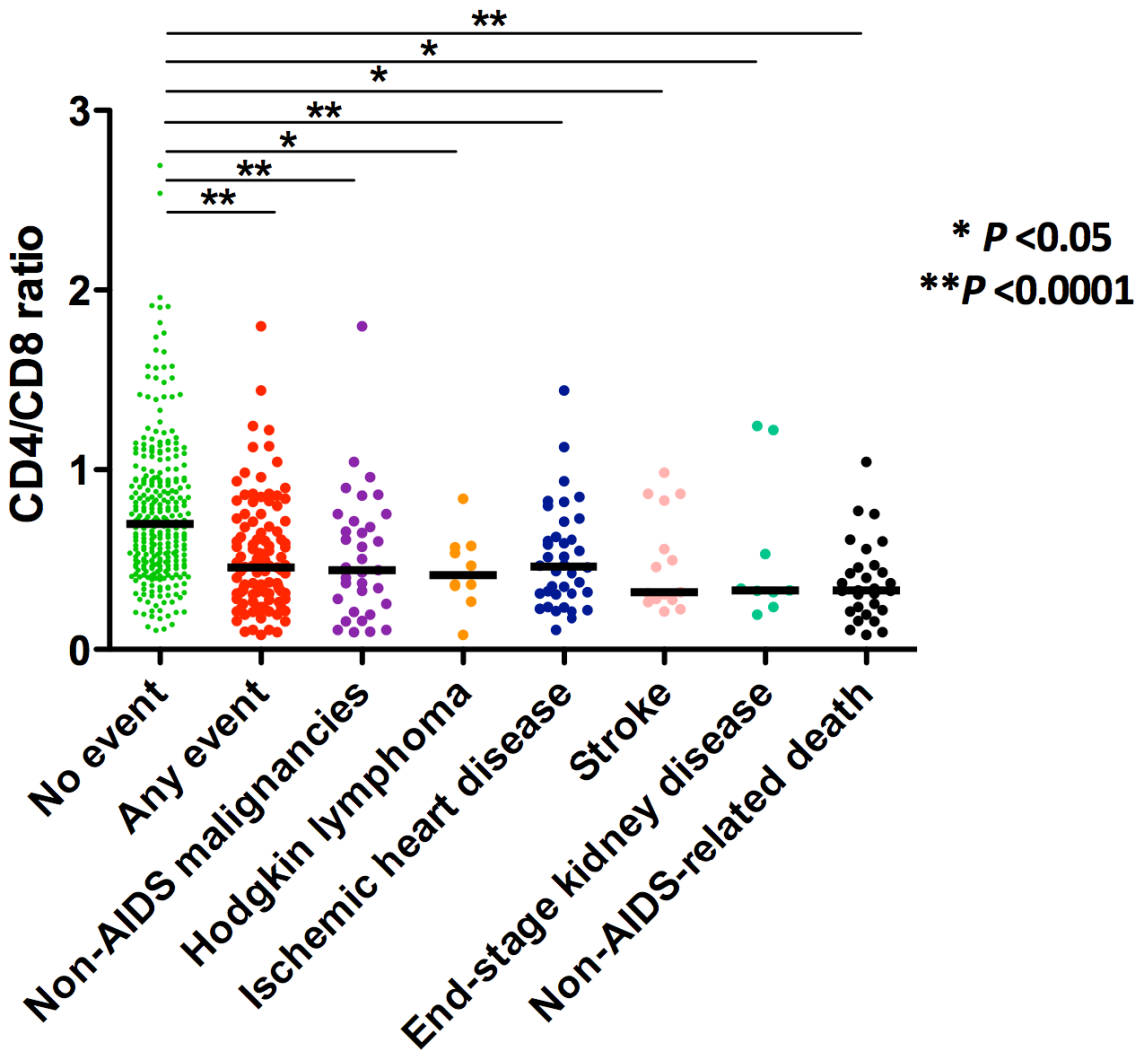
CD4/CD8 ratio according to the presence and type of event. The CD4/CD8 in controls [0.70 (IQR 0.48–0.94)] was significantly higher than in subjects who developed non-AIDS events [0.46 (IQR 0.31–0.68), *P*<0.0001], including non-AIDS defining malignancies (N = 35) [0.44 (IQR 0.25–0.71), *P* = 0.0001], Hodgkin lymphoma (N = 10) [0.41 (IQR 0.33–0.57), *P = *0.0039], ischemic heart disease (N = 38) [0.47 (IQR 0.32–0.63), *P* = 0.0001], stroke (N = 15) (0.46 [IQR 0.28–0.83), *P* = 0.013), and end-stage kidney disease (N = 9) [0.33 (0.32–0.53), P = 0.028]. The CD4/CD8 ratio was also significantly lower in subjects with non-AIDS associated mortality (N = 29) [0.33 (IQR 0.22–0.46), *P*<0.0001].

**Figure 2** illustrates the CD4/CD8 ratio in controls and in cases across the different types of events (for a description of the events, see **Table 2**). The CD4/CD8 in controls [0.70 (IQR 0.48–0.94)] was significantly higher compared to the ratio in subjects who developed non-AIDS events [0.46 (IQR 0.31–0.68), P<0.0001], including non-AIDS defining malignancies (N = 35) [0.44 (IQR 0.25–0.71), P = 0.0001], Hodgkin lymphoma (N = 10) [0.41 (IQR 0.33–0.57), P = 0.0039], ischemic heart disease (N = 38) [0.47 (IQR 0.32–0.63), P = 0.0001], stroke (N = 15) (0.46 [IQR 0.28–0.83), P = 0.013), and end-stage kidney disease (N = 9) [0.33 (0.32–0.53), P = 0.028]. The CD4/CD8 ratio was also significantly lower in subjects with non-AIDS associated mortality (N = 29) [0.33 (IQR 0.22–0.46), P<0.0001].

**Table 2:** Description of non-AIDS-related events.

Type of Event	No. (%)	Death (No., %)
**Cardiovascular**	**55 (50.5)**	**27 (29.6)**
Ischemic Heart Disease	38 (34.9)	5 (18.5)
Stroke	15 (15.6)	3 (11.1)
**End-stage renal disease**	**9 (8.3)**	**4 (14.8)**
**Non-AIDS-related neoplasias**	45 (42.1)	
Pulmonary	10 (9.2)	8 (29.6)
Gastrointestinal	10 (9.2)	4 (14.8)
Urologic	5 (4.6)	1 (3.7)
Hematologic		
*Hodgkin lymphoma*	10 (9.2)	1 (3.7)
*Castleman ‘s Disease*	2 (1.8)	–
Eyes, ears, nose or throat	4 (3.7)	1 (3.7)
Breast	3 (2.8)	–
Skin (melanoma)	1 (0.9)	–
**Total**	**109 (100)**	**27 (100)**

### Relations with the CD4/CD8 Ratio

We analyzed the associations between the study variables and the CD4/CD8 ratio. The CD4/CD8 ratio showed weak albeit statistically significant correlations with age (Rho −0.152, P = 0.001), nadir CD4 (Rho −0.20, P<0.001) and cumulative ART exposure (Rho 0.14, P = 0.042) and was significantly lower in men compared to women (0.71 Vs. 0.61, P = 0.032).

### Discriminatory Ability of the CD4/CD8 Ratio for the Prediction of non-AIDS Events

The discriminatory ability of the CD4/CD8 ratio, CD4+ T-cell count, CD8+ T-cell count and nadir CD4 was explored by obtaining the AUC. The CD4/CD8 ratio showed the best AUC (0.720, 95% CI 0.662–0.777), significantly higher than that for CD4+ T-cell count (0.671), CD8+ T-cell count (0.435) or nadir CD4 (0.662). The most accurate cutoff of the CD4/CD8 ratio for the detection of non-AIDS events in a sensitivity/specifity plot was 0.4, with a sensitivity of 0.83 and a specificity of 0.45.

### Associations between the CD4/CD8 Ratio and non-AIDS Related Morbidity and Mortality

Univariate and multivariate analyses are summarized in **Table 3**. In the univariate analysis, a lower CD4/CD8 ratio was associated with increased risk of non-AIDS events (per quartile decrease; OR, 2.0; 95% CI, 1.6–2.5). In the multivariate analysis, after adjusting by gender, age, nadir CD4, date of ART initiation and cumulative ART exposure, subjects with lower CD4/CD8 ratio displayed greater risk of non-AIDS events (per quartile decrease; OR 2.6; 95% CI, 1.7–4.0; for CD4/CD8 ratio <0.4; OR, 5.1; 95% CI, 2.3–12.4). Following the same strategy, a low CD4/CD8 ratio was a predictor of non-AIDS mortality (per quartile decrease; OR, 2.8; 95% CI, 1.5–5.3; for CD4/CD8 ratio <0.4; OR, 4.5; 95% CI, 1.7–11.8).

**Table 3:** Risk of serious non-AIDS events associated with a low CD4/CD8 ratio (categorized by quartiles and by the cut-off of 0.4).

Explanatory logistic regression(All sample)	4^th^ Qrt.	3^rd^ Qrt.		2^nd^ Qrt.		1^st^ Qrt.		OR per Qrt. Decrease		CD4/CD8 ratio <0.4	
		OR (95% CI)	P	OR (95% CI)	P	OR (95% CI)	P	OR (95% CI)	P	OR (95% CI)	P
#Cases/Controls	11/91	19/83		31/71		48/53		109/298		109/298	
Unadjusted	1.0	1.89 (0.85, 4.21)	0.118	3.61 (1.69, 7.68)	0.001	7.49 (3.58, 15.66)	<0.0001	1.96 (1.57, 2.45)	<0.0001	4.79 (2.91, 7.90)	<0.0001
Adjusted*	1.0	2.57 (0.63, 10.54)	0.188	10.61 (2.78, 40.50)	0.001	15.77 (3.65, 68.18)	<0.0001	2.56 (1.66, 3.94)	<0.0001	5.11 (2.31, 12.42)	<0.0001
#Cases/Controls in subjects withnadir <200	4/24	13/35		19/39		34/41		70/139		70/139	
Unadjusted	1.0	2.23 (0.65, 7.66)	0.204	2.92 (0.89, 9.62)	0.078	4.98 (1.57, 15.74)	0.006	1.61 (1.19, 2.17)	0.002	2.91 (1.59, 5.35)	0.001
Adjusted*	1.0	1.45 (0.14, 15.09)	0.754	6.82 (0.75, 62.30)	0.089	15.49 (1.53, 156.16)	0.020	2.77 (1.41, 5.42)	0.030	7.90 (2.37, 26.38)	0.001
#Cases/Controls in subjects withCD4>350	10/86	15/75		17/64		17/18		59/243		59/243	
Unadjusted	1.0	1.72 (0.73, 4.05)	0.215	2.28 (0.98, 5.32)	0.055	8.12 (3.19, 20.62)	<0.0001	1.88 (1.40, 2.53)	<0.0001	7.16 (3.24, 15.83)	<0.0001
Adjusted*	1.0	1.48 (0.14, 15.55)	0.742	8.40 (0.92, 76.06)	0.058	15.03 (1.49, 150.75)	0.021	2.67 (1.38, 5.16)	0.003	6.57 (2.04, 21.14)	0.002
**Conditional logistic regression** **(Matched analysis)** †	**4^th^ Quartile**	**3^rd^ Quartile**		**2^nd^ Quartile**		**1^st^ Quartile**		**OR per Quartile Decrease**		**CD4/CD8 ratio <0.4**	
		**OR (95% CI)**	**P**	**OR (95% CI)**	**P**	**OR (95% CI)**	**P**	**OR (95% CI)**	**P**	**OR (95% CI)**	**P**
#Cases/Controls	17/33	25/25		24/26		34/16		100/100		100/100	
Primary	1.0	2.45 (1.02, 5.86)	0.044	2.28 (0.92, 5.67)	0.076	11.46 (3.20, 40.97)	<0.0001	1.95 (1.32, 2.73)	<0.0001	3.43 (1.48, 7.96)	0.004
Adjusted§	1.0	3.04 (0.45, 20.43)	0.252	3.56 (0.83, 32.57)	0.084	31.99 (2.70, 378.85)	0.006	2.89 (1.32, 6.17)	0.007	5.43 (1.89, 26.99)	0.004

Seven subjects from the 407 individuals were not included in the multivariate analysis due to missing data on CD4 nadir/cumulative ART exposure.

* Multivariate analysis adjusted by age, gender, nadir CD4+ cell count, date of ART initiation and cumulative ART exposure.

† Case-control substudy matched by age, gender, nadir CD4+ cell count and proximal CD4+ cell count.

§ Conditional logistic regression analysis adjusted for date of ART initiation and cumulative ART exposure.

To assess whether the CD4/CD8 ratio might maintain the independent association with non-AIDS events among subjects with low nadir CD4, we re-applied the model in the subgroup of patients with nadir CD4 below 200 cells/µL (N = 212) and the CD4/CD8 ratio remained independently associated to non-AIDS events (per quartile decrease; OR 2.8; 95% CI 1.2–2.2). Also, we analyzed whether the CD4/CD8 ratio may remain independently associated to non-AIDS events in subjects with successful immunological response to ART by re-applying the model in the subgroup of patients with CD4 counts >350 cells/µL (N = 298), and subjects with low CD4/CD8 ratio displayed increased risk of non-AIDS events (per quartile decrease, OR, 6.6; 95% CI, 2.0–21.1).

In the conditional logistic regression in the group of 200 subjects matched by age, gender, nadir CD4 and CD4+ T-cell count and adjusted by date of ART initiation and cumulative ART exposure, we observed comparable associations between the CD4/CD8 ratio and non-AIDS events (per quartile decrease; OR, 2.9; 95% CI, 1.3–6.1; for CD4/CD8 ratio <0.4; OR, 5.4; 95% CI, 1.9–27.0).

## Discussion

In this study in ART-treated HIV-infected patients, a low CD4/CD8 ratio was strongly associated with the risk of non-AIDS morbidity and mortality. Remarkably, the CD4/CD8 ratio was consistently lower across all types of non-AIDS events included in the definition (ischemic heart disease, stroke, end-stage kidney disease, and non-AIDS malignancies) and performed better than the CD4+ T-cell count, CD8+ T-cell count or nadir CD4 to discriminate subjects at risk of serious non-AIDS events. Noteworthy, this association hold robustly and was independent of nadir CD4 and proximal CD4+ T-cell counts, as it was even stronger in the subgroup of patients with low nadir CD4, as well as in those with higher CD4+ T-cell counts, or in a subpopulation of 200 cases and controls matched by age, gender, nadir CD4 and CD4+ T-cell count.

In our view, the importance of the HIV viral load and CD4+ T-cell count in the clinical management of HIV-infected subjects and as predictors of clinical outcomes left little room in past years for any other clinically useful marker. The CD4/CD8 ratio has proved to be useful in a number of areas. In the setting of HIV infection, this ratio was identified before the introduction of the highly active ART in 1996 as a predictor of development of AIDS [33]. More recently, in a study in the Swiss cohort a low CD4/CD8 ratio before initiation of ART was a predictor of Hodgkin lymphoma [25], and in a case-control study the CD4/CD8 ratio was independently associated with subclinical atherosclerosis [24], which in turn has been associated with immune activation and inmmunosenescence [34], as most of non-AIDS-associated conditions [10]. These observations provided support to speculate that the CD4/CD8 ratio might be a marker of non-AIDS-related diseases in treated HIV-infected patients. Outside HIV infection, a low CD4/CD8 ratio, namely the immune risk profile, has been proposed as a surrogate marker of the collection of immune-related defects that defines “immunosenescence” –a phenomenon characterized by the T-cell proliferation and differentiation resulting in the generation of antigen experienced, highly differentiated and dysfunctional T-cells [13]– and correlated with all-cause mortality in the elderly [18]–[20], [35]. Given these studies, we recently evaluated the biological and clinical significance of the CD4/CD8 ratio in treated HIV infection. First, in an exploratory study in long-term virally suppressed adults we found that the CD4/CD8 ratio inversely correlated with activated CD4+ (HLADR+CD38+) and CD8+ (HLADR+) T-cells [21]. Then, in 38 vertically HIV-infected children and adolescents on ART we observed independent associations between the CD4/CD8 ratio and CD8+ T-cell activation (HLADR+CD38+, HLADR+PD1-) and senescence (CD57+CD28−) [22]. Subsequently, we found in 132 HIV-infected subjects with long-term viral suppression and CD4+ >350 cells/µL that a low CD4/CD8 ratio identified individuals with markers of age-associated disease (higher carotid intima-media thickness, higher arterial stiffness, and lower estimated glomerular filtration rate) [23].

These observations are physiopathologically supported by the fact that non-AIDS-related illnesses are mainly a consequence of the increased burden of age-associated disease and likely to be driven, at least in part, by persistent immune activation [10]. Levels and recurrence of T-cell activation is a major driving a factor of immunosenescence, which in turn have been associated with the CD4/CD8 ratio both in the general population [20] and in HIV-infected individuals [21], [22]. While in healthy subjects these immune responses as a result of immune activation are followed by replenishment of the CD8+ T-cell pool from naïve CD8+ T-cells [13], HIV-infected subjects show an impaired capacity to replace senescent T-cells, most probably due to reduced thymic output, which causes decreased levels of naïve CD8+ T-cell and a compensatory expansion of memory CD8+ T-cells [13], [36], [37]. Since among immunological ART responders a low CD4/CD8 ratio is driven by very high numbers of CD8+ T-cells, this physiopathological cascade may explain why in this situation the risk of non-AIDS associated morbidity and mortality might be markedly increased, as they probably represent a population with expanded activated/senescent CD8+ T-cells. In our view, this particular population of immunological responders with low CD4/CD8 ratio –hence, very high CD8+ T-cells– could represent a clinical phenotype of immunological responders at higher risk for non-AIDS-associated morbidity and mortality.

Strengths of this study include the use of a prospective cohort subject to external audit of data and the ascertainment of serious non-fatal and fatal non-AIDS events, as well as the inclusion of a matched case/control subanalysis. There are also some limitations. Since we restricted the definition of non-AIDS events to ischemic heart disease, stroke, end-stage kidney disease, and non-AIDS malignancies, we cannot provide information on the value of the CD4/CD8 ratio to identify subjects at higher risk of other non-AIDS conditions. Also, we did not adjust the analyses by other potential risk factors for non-AIDS-related diseases. However, to date, no factor in the general population apart of age, gender and CMV serostatus have proved to impact on the CD4/CD8 ratio and our main objective was to analyze could provide additional information to nadir CD4 and CD4+ T-cell counts to identify subjects at increased risk of serious non-AIDS events. Also, cases showed significantly longer cumulative ART exposure than controls, in both the general sample and the nested matched study. Although we controlled the analyses for this variable, it is important to note that this difference between groups might have led to underestimate the association of a low CD4/CD8 ratio with non-AIDS-associated morbidity and mortality, given that in a recent study the CD4/CD8 ratio appeared to increase still after more than ten year of ART-mediated HIV RNA suppression [38], and in our study subjects who developed serious non-AIDS-related illnesses showed a lower CD4/CD8 ratio despite longer cumulative ART exposure. Lastly, data were retrieved from a single cohort of HIV-infected individuals, so before using the CD4/CD8 ratio as a surrogate of serious non-AIDS-related illnesses, these results should be reproduced in larger and prospective studies.

These findings have potential clinical implications, since patients with failure to increase the CD4/CD8 ratio despite achieving full immunovirological response to ART might benefit from screening programs and aggressive management of concomitant risk factors for age-associated disease. Moreover, considering previous data on the CD4/CD8 ratio in the general population [17], [19], [20] and our previous studies in HIV-infected individuals [21], [22] subjects with low CD4/CD8 ratio despite otherwise successful ART might be of outstanding interest to be included in clinical trials aiming to reduce chronic immune activation. Of importance, since the CD4/CD8 ratio is available in routine clinical practice, its use as a predictor of non-AIDS associated morbidity and mortality might be easily implemented in clinical settings.

In conclusion, our results suggest that a low CD4/CD8 ratio might identify a subset of individuals at increased risk of non-AIDS-associated morbidity and mortality. The association between this ratio and the risk of serious non-AIDS events is robust and maintained within subjects with low CD4 nadir or those with higher CD4+ T-cell count.
